# Biomarkers for hepatocellular carcinoma: progression in early diagnosis, prognosis, and personalized therapy

**DOI:** 10.1186/2050-7771-1-10

**Published:** 2013-02-05

**Authors:** Kai Zhu, Zhi Dai, Jian Zhou

**Affiliations:** 1 Liver Cancer Institute, Zhongshan Hospital, Fudan University, Shanghai, 200032, China; 2 Key Laboratory of Carcinogenesis and Cancer Invasion, Fudan University, Ministry of Education, Shanghai, 200032, China; 3 Shanghai Key Laboratory of Organ Transplantation, Zhongshan Hospital, Fudan University, Shanghai, 200032, China

**Keywords:** Hepatocellular carcinoma, Early diagnosis, Prognosis, Biological markers

## Abstract

Hepatocellular carcinoma (HCC) is one of the most common malignant tumors in the world. Currently, surgical resection, liver transplantation, and local ablation are considered curative therapeutic practices for HCC. The diagnosis of HCC without pathologic confirmation is achieved by analyzing serum alpha-fetoprotein (AFP) levels combined with imaging techniques, including ultrasonography, magnetic resonance imaging, and computerized tomography. Although progress has been made in the diagnosis and management of HCC, its prognosis remains dismal. Various new technologies have identified numerous novel biomarkers with potential diagnostic as well as prognostic value, including Dickkopf-1 and Golgi protein 73. These biomarkers not only help in the early diagnosis and prediction of prognosis, but also assist in identifying potential targets for therapeutic interventions. In this article, we provide an up-to-date review of the biomarkers that are used for early diagnosis, prognosis prediction, and personalized treatment of HCC.

## Introduction

Hepatocellular carcinoma (HCC) is one of the most frequently diagnosed cancers worldwide. The disease is predominant in Asia and Africa, but its incidence is steadily increasing throughout the rest of the world [1]. Most HCC develop in patients with a history of chronic hepatitis or cirrhosis in which there is continuous inflammation and regeneration of hepatocytes. Unlike other solid malignancies, the coexistence of inflammation and cirrhosis makes the early diagnosis and prognostic assessment of HCC much more difficult. This complication highlights the need to identify valuable biomarkers for the diagnosis and treatment of HCC.

The proliferation and survival of cancer cells require a process called oncogene addiction, which is the activation of specific oncogenes and inactivation of specific tumor suppressors, such as Rb1 in retinoblastoma [2] and BRCA1 in breast cancer [3]. However, no specific oncogene addictions have been observed in HCC, which is a complex disease with a variety of underlying pathogenic anomalies caused by multiple risk factors. The lack of ideal biomarkers for HCC diagnosis, prognosis, and therapy has posed a major challenge to HCC management.

With advances in the understanding of tumor biology, interest in identifying molecular biomarkers of HCC has increased. Over the last decade, a number of new cutting-edge technologies such as next-generation sequencing [4,5] and microarray technologies [6-8] have emerged, leading the search for biomarkers into a new era of “omics” [9,10]. Using these technologies, it is now quite easy to examine a whole tumor genome (including copy number variations, loss of heterogeneity, aneuploidy, single nucleotide polymorphism) [11-14], transcriptome [15,16], proteome [17,18], epigenome [19,20], metabolome [21-23], and miRNA profile [24,25], and the analysis of tens of thousands of molecular targets has become affordable and operable. Currently, numerous circulating markers and tissue markers have been identified [17,26-30]; however, few biomarkers are acceptable for clinical utility because of their low predictive accuracy and/or high cost. Here, we provide an up-to-date review of the biomarkers that are used for early diagnosis, prognosis, and personalized treatment of HCC.

## Review

### Biomarkers for early diagnosis

The diagnosis of HCC without pathologic confirmation can be achieved by assessing the serum alpha-fetoprotein (AFP) level combined with imaging techniques, including ultrasonography, magnetic resonance imaging, and computerized tomography [31,32]. However, improvement in early diagnosis is still needed because only 44% of the patients are diagnosed at a localized disease stage, and only 30% of patients with HCC are candidates for potentially curative treatments at the time of diagnosis [33]. Thus, the discovery of an effective, reliable tool for early diagnosis of HCC to increase the number of patients who are suitable for curative treatment will play a pivotal role in improving HCC patients’ prognosis.

A marker for early diagnosis would meet the following requirements: first, it should achieve high accuracy, which would increase the probability of a diagnosis being made prior to spread and thus increase the cure rate; second, specimen collection for detecting the marker should be easily operable and non-invasive; and third, the cost-effectiveness should be considered [34]. Tumor tissue-oriented markers are not highly practical because not all tumor tissues can be obtained at an early stage and the invasive procedure may cause spread of tumor cells. Biomarkers from body fluids such as serum, plasma, urine, and bile are suitable candidates for early diagnosis of HCC because they are easily accessible [35]. In the following section, we list some important circulating (serum or plasma) markers for early diagnosis of HCC.

#### Protein

Since AFP was discovered in the serum of HCC patients in 1964 [36], it has been regarded as the most useful serum protein thus far for patients at risk for HCC [37-39]. However, its sensitivity for detecting HCC ranges between 25%-60% [39,40], and its specificity is also low because serum AFP can also be detected in patients with cirrhosis (11%-47%) and chronic hepatitis (15%-58%).

In addition to AFP, more than 20 serum proteins have clinical significance in early diagnosis of HCC [10,41], among which several proteins are proved to have advantages over AFP.

##### DKK1

DKK1 belongs to a family of secreted proteins that play an important role in HCC progression through the promotion of cytoplasmic/nuclear accumulation of beta-catenin in HCC cells via the Wnt/beta-catenin signaling pathway [42].

Recently, Shen et al. [41] reported that serum DKK1 is a promising candidate for HCC diagnosis. The authors retrospectively assessed serum DKK1 in 1284 patients (633 with HCC, 171 with chronic HBV infection, 168 with cirrhosis, and 312 healthy controls) and found that DKK1 has better diagnostic value for HCC than does AFP, especially for patients with AFP-negative and early stage HCC. Combined testing of serum DKK1 and AFP concentrations improved diagnostic accuracy for HCC versus all controls compared with either test alone. Nevertheless, DKK1 is not overly specific for HCC diagnosis, and a recent study reported that serum DKK1 was also elevated in patients with intrahepatic cholangiocarcinoma [43].

##### Golgi protein 73 (GP73)

GP73 is a 73 kDa trans-membrane glycoprotein that normally resides within the Golgi complex. It is expressed in normal biliary epithelial cells whereas normal hepatocytes do not express this protein, and its expression is significantly increased in liver diseases such as HCC [44].

Serum GP73 is a valuable biomarker for patients with HCC [45,46]. Mao et al. [46] compared serum GP73 and AFP in 4217 participants, including 1690 healthy adults, 337 HBV carriers, 512 patients with cirrhosis, 789 patients with HCC, 61 patients with other malignant liver lesions, 206 patients with benign liver lesions and 622 patients with 14 non-liver cancers. The sensitivity and specificity of serum GP73 for HCC were 74.6% and 97.4%, respectively, compared with 58.2% and 85.3% for AFP. The GP73 level significantly increased in patients with HCC compared with healthy controls, decreased following surgical resection of HCC lesions and increased with tumor recurrence. Although the control group included HBV carriers, this group lacked patients with chronic hepatitis, whereas most HCC patients have hepatitis.

##### Protein induced by vitamin K absence or antagonist II (PIVKA-II)

PIVKA-II, an abnormal prothrombin discovered in 1984, has been widely proposed to be a useful HCC biomarker [47]. Takikawa et al. [48] measured plasma levels of PIVKA-II and AFP in 628 patients with various diseases, including 253 patients with liver cirrhosis and 116 patients with HCC. PIVKA-II was detected in 54.3% of patients with HCC, and the concentration showed a positive correlation with the tumor size. As a screening test for detecting HCC, PIVKA-II yielded sensitivity and specificity values (52.8% and 98.8%, respectively) that were comparable with AFP. Beale et al. [49] assessed AFP and PIVKA-II levels in pre-treatment serum samples from 50 patients with HCC, and the combination of serum AFP and PIVKA-II was better for detecting HCC than using either AFP or PIVKA-II alone.

#### Nucleic acids

Nucleic acids, including DNA, RNA, and nucleosomes, can be detected in the circulation of patients with HCC, and changes in their levels have been associated with tumor burden and progression of malignancy [50]. In the past decade, circulating nucleic acids have been extensively studied with regard to their diagnostic significance [51-54]. For instance, plasma AFP mRNA [28,55] is considered to be a diagnostic marker for HCC. Accumulating evidence has shown that microRNAs (miRNAs) play important roles in cancer initiation, propagation, and progression [56-58]. MiRNA deregulation occurs at early stages of HCC and increases throughout the various steps of hepatocarcinogenesis [52]. There are multiple studies on the diagnostic function of miRNA in HCC diagnosis [52,54,59]. However, the diagnostic value of miRNAs is limited by one or more of the following factors: limited number of screened miRNAs, small sample size, failure to differentiate HCC from hepatitis, and lack of independent validation.

Recently, we measured plasma miRNA expression profiles (723 miRNAs) in a large cohort of 934 participants that included healthy individuals and patients with chronic HBV infection, cirrhosis, or HBV-related HCC. We identified a miRNA panel (miR-122, miR-192, miR-21, miR-223, miR-26a, miR-27a, and miR-801) that provided high diagnostic accuracy for discriminating patients with HCC from the healthy population (AUC = 0.941) and patients with chronic HBV (AUC = 0.842) or cirrhosis (AUC = 0.884). This finding led to the conclusion that the plasma miRNA panel had considerable clinical value for the early diagnosis of HCC and could help patients who might have otherwise missed the curative treatment window benefit from optimal therapy [54].

### Prognostic biomarkers

Surgical resection, liver transplantation and local ablation are considered curative therapeutic practices for HCC. Other modalities, such as targeted therapy and transarterial chemoembolization (TACE), are palliative treatments. Despite these curative or palliative treatments, prognosis is still poor due to underlying liver diseases and the unique biology of HCC. As a result, biomarkers that better predict patients who are at higher risk of recurrence and poorer prognosis would help guide their treatment [26].

A number of biomarkers have been reported to predict the outcome of these therapies, including CD151 and CXCL5 for surgical treatment [27,60], AFP and LDH for TACE [61,62], PIVKA-II and VEGF for radiofrequency ablation (RFA) [63,64], and serum AFP and HBeAg for percutaneous ethanol injection (PEI) [65-67].

#### Biomarkers for surgical treatment

Surgical treatment offers a potentially curative option for HCC patients, but patients’ outcomes are varied due to differing tumor characteristics. Additionally, the exact biology of HCC remains poorly understood, thus making prediction of outcome after surgical resection very difficult. The prognosis of HCC patients does not simply reflect the size and number of the tumors; instead, prognosis is affected by a complex interplay between known and unknown factors, including tumor biology, patient condition, etc. [35]. Thus, the ability to predict which patients have a poor prognosis would help to assign risk and guide surgery and other treatments.

##### Circulating biomarkers

Circulating biomarkers are still preferred for prognostic prediction because they are easily accessible. Serum AFP is commonly used for diagnosis and surveillance of HCC [37,39] and has been suggested as an independent indicator for prognosis. HCC patients with a high serum AFP level tend to have shorter survival [38,53].

Other circulating factors such as Ang2 [53], VEGF [53,68,69], HGF [70,71], and TGF-beta [72], are also independent factors for HCC prognosis. A recent study proposed that plasma macrophage migration inhibitory factor (MIF) levels have prognostic value in HCC patients. Plasma MIF levels have a significant association with overall survival (OS) and disease-free survival (DFS) of HCC patients, even in patients with normal serum AFP levels and Tumor Node Metastasis (TNM) stage I HCC [73].

Circulating tumor cells (CTCs) may reflect tumor aggressiveness and serve as a promising candidate for predicting tumor recurrence and metastasis [74]. However, their utility is limited due to the rarity of CTCs in peripheral blood of the patients. Recent technical advances have made it possible to detect CTCs; therefore, their clinical value has been tested in multiple tumor types, including breast cancer [75], lung cancer [76], and prostate cancer [77]. Sun et al. proved that EpCAM-positive CTCs may serve as a prognostic marker in HCC after curative resection [78].

##### Tumor tissue biomarkers

Research into tumor tissues can provide direct biological information about the tumors; thus, the search for tumor biomarkers is crucial. A plethora of HCC tumor cell-derived biomarkers with potential prognostic significance have been identified in recent decades [9,17,26,35,79-81], but consensus could not be reached.

HCC-related proteins have been extensively explored for use in determining prognosis [9-11,82]. For instance, our previous study investigated CXCL5 (epithelial neutrophil-activating peptide-78) expression in a large cohort of 919 HCC patients. The results showed that overexpression of CXCL5 was well correlated with intratumoral neutrophil infiltration and that CXCL5 overexpression alone or in combination with the presence of intratumoral neutrophils was an independent prognostic indicator for OS and cumulative recurrence in HCC patients [60]. In addition, our institute also searched extensively for prognostic biomarkers in HCC patients undergoing liver transplantation [17,83]. By investigating tumor tissues of 232 HCC patients, we identified calpain small subunit 1 (Capn4) as an independent prognostic factor for recurrence and survival in HCC patients after liver transplantation [17].

Cancer stem cells (CSCs) may play a pivotal role in the progression of tumors [84,85]. CSCs represent the tumorigenic cells that generate tumors via the stem cell processes of self-renewal and differentiation. CSCs may persist in tumors as a distinct population and cause relapse and metastasis by giving rise to new tumors [86]. Although the existence of CSCs in HCC is still controversial, several studies have demonstrated the clinical significance of CSC markers in HCC patients [10,79]. These markers include CD90 [87], CD133 [29], CD13 [88], and EpCAM [89].

The role of the microenvironment surrounding tumor cells for the initiation and progression of HCC is becoming increasingly clear [30,90-92]. The tumor microenvironment, also named the tumor stroma, includes the extracellular matrix (ECM) and all other non-tumor cell types within a tumor tissue (e.g. endothelial cells, fibroblasts, and cells of the immune system). Various tumor stroma-associated factors, such as regulatory T cells (Tregs) [93], macrophage colony-stimulating factor (M-CSF) [94], macrophages [95], and hepatic stellate cells [96], have been investigated and exhibit significant prognostic value. For instance, Budhu et al. [97] showed that a unique inflammation/immune response-related signature in the venous metastasis-associated liver microenvironment coincides with elevated expression of M-CSF and can serve as a superior predictor of HCC venous metastases when compared with other clinical prognostic parameters.

#### Biomarkers for TACE

Although patients with early stage HCC have the chance to undergo curative treatment, most HCC patients are still diagnosed at a late stage when curative treatment is no longer applicable. For these patients, based on randomized, controlled clinical trials, TACE may be an effective treatment option for reducing systemic toxicity, increasing local antitumor effects, and improving survival [98,99]. However, there are markedly diverse outcomes after TACE in terms of treatment response and survival. Therefore, identifying markers that can predict TACE treatment outcomes before choosing this treatment option is an important endeavor.

The most promising prognostic candidates for TACE are circulating biomarkers. Some studies have reported that serum AFP [61], circulating nucleosomes [100], blood neutrophil-to-lymphocyte ratio [101], and lactate dehydrogenase [62], are prognostic factors for TACE. As an example, Wang et al. [61] retrospectively studied the survival of 441 HCC patients (including 139 patients with normal AFP levels and 302 patients with elevated AFP levels) after TACE, and found that patients with normal AFP levels had a better treatment response and prognosis after TACE than patients with elevated AFP levels.

### Personalized therapy

The recent discovery of new therapeutic targets based on the molecular pathways that are involved in hepatocarcinogenesis has led to exciting results in targeted treatment of HCC patients. Investigators have attempted to select therapeutic options for patients according to their tumor’s molecular profile, and this treatment modality will pave the way for personalized treatment of HCC.

#### Targeted therapy

Targeted therapy that specifically inhibits molecular abnormalities has emerged as an effective therapeutic option for malignancies [102,103]. Small molecule tyrosine kinase inhibitors have great potential for the treatment of HCC through targeting several growth factors and their associated signaling pathways (e.g. EGF/EGFR, VEGF/VEGFR, IGF/IGFR, PDGF, FGF, RAS/RAF/ERK/MAPK, PI3K/AKT/mTOR, Wnt/beta-catenin) [104,105]. Currently, nearly 60 reagents are being investigated for treatment of HCC, but only sorafenib have been proven effective in patients with advanced HCC [106].

Sorafenib is an oral multi-kinase inhibitor that competitively inhibits ATP binding to the catalytic domains of various kinases, such as Raf kinase, VEGFR-2, -3, and PDGFR, thereby increasing apoptosis and decreasing angiogenesis and cell proliferation [79,106,107]. However, no specific marker can guide the use of sorafenib in HCC; in contrast, HER2 and EGFR expression can positively predict the therapeutic response rate of trastuzumab in breast cancer and cetuximab in non-small cell lung cancer, respectively.

Other oral tyrosine-kinase inhibitors including sunitinib, linifanib, brivanib, and regorafenib block a number of angiogenesis-related signaling pathways, such as VEGFR, PDGFR, and FGFR [35,104,107]. Although many clinical trials have been discontinued because of poor effectiveness or severe adverse effects, these approaches provide critical insight into the mechanisms of targeted therapy for HCC and may finally allow us to optimize the current therapies for this fatal disease.

#### Interferon-alpha

Interferon-alpha is a multifunctional cytokine that postpones recurrence of HCC and improves OS in HCC patients after curative resection [108-110]. However, the benefit of interferon-alpha therapy is usually modest because it is not effective for all patients, and it is difficult to determine which patients will respond well to interferon-alpha [108,111]. A recent study analyzed the miRNA profiles of 455 patients with HCC who had undergone curative tumor resection and assessed the association of the miRNA profiles with survival and response to therapy with interferon-alpha. The study showed that HCC patients whose tumors express low levels of miR-26 have a better response to interferon-alpha therapy than patients with high levels, suggesting that miR-26 expression status could be used as a predictor of the response to interferon-alpha therapy [112]. At present, a multicenter, randomized controlled trial assessing the impact of low miR-26 expression on interferon-alpha adjuvant therapy for HCC patients is ongoing in China (NCT01681446, http://clinicaltrials.gov/ct2/show/NCT01681446?term=jia+fan&rank=3).

## Conclusions

New technologies have identified numerous novel biomarkers with potential diagnostic and prognostic value. Recent advances in identification, isolation, and capture of tumor-derived microvesicles will reveal new insights into HCC diagnosis and personalized therapy [113]. Nevertheless, most of these markers have been studied retrospectively; few prospective trials have evaluated their clinical significance, or clinical application.

Because HCC is a complex disease with multiple underlying pathogenic mechanisms caused by a variety of risk factors, it is difficult to characterize HCC with a single biomarker. Thus, signatures of a combination of biomarkers may be more valuable for the diagnosis, staging, and prognosis of HCC. In the near future, identifying non-invasive and cost-effective biomarkers for early diagnosis and personalized treatment of HCC will be one of the most promising fields of biomarker research.

## Abbreviations

HCC: Hepatocellular carcinoma; AFP: Alpha-fetoprotein; GP73: Golgi protein 73; PIVKA-II: Protein induced by vitamin K absence or antagonist II; miRNA: microRNA; TACE: Transarterial chemoembolization; RFA: Radiofrequency ablation; PEI: Percutaneous ethanol injection; MIF: Migration inhibitory factor; OS: Overall survival; DFS: Disease-free survival; TNM: Tumor Node Metastasis; CTC: Circulating tumor cell; Capn4: Calpain small subunit 1; CSC: Cancer stem cell; ECM: Extracellular matrix; Treg: Regulatory T cell; M-CSF: Macrophage colony-stimulating factor.

## Competing interests

The authors declare that they do not have anything to disclose regarding funding from industries or conflict of interest with respect to this manuscript.

## Authors’ contributions

ZK drafted the manuscript. All authors read and approved the final manuscript.

## Grant support

This study was jointly supported by the National Key Sci-Tech Special Project of China (Grant No. 2012ZX10002-016), National Science Fund for Distinguished Young Scholars (81225019) and National Natural Science Funds of China (No. 812250125; No. 81172277; No. 81272724).
